# Influence of community-based education on undergraduate health professions students’ decision to work in underserved areas in Uganda

**DOI:** 10.1186/s13104-017-3064-0

**Published:** 2017-12-08

**Authors:** Samuel Kizito, Rhona Baingana, Kintu Mugagga, Peter Akera, Nelson K. Sewankambo

**Affiliations:** 1 Makerere University College of Health Sciences, Kampala, Uganda; 2 School of Health Sciences, Kampala International University, Kampala, Uganda; 3 Faculty of Medicine, Gulu University, Gulu, Uganda; 4 Clinical Epidemiology Unit, School of Medicine, College of Health Sciences, Makerere University, Kampala, Uganda

**Keywords:** Doctor, Mal-distribution, Rural areas, Uganda

## Abstract

**Background:**

Uganda is beset by a shortage of health workers and the few available are mal-distributed. Providing rural exposure through community-based education could positively influence students’ perspectives towards work in rural areas. We aimed to assess the impact of Community-Based Education and Research (COBERS) on health professions students’ attitudes towards working in rural areas. This was a before-and-after study among 525 students of 4 medical universities in Uganda. Data was collected using self-administered paper-based questionnaires. Logistic regression and Poisson regression respectively were used to assess intention and intended number of years of work in rural areas.

**Results:**

Before COBERS, 228/518 (44.0%) students indicated that they intended to work in rural areas as compared to 245/506 (48.4%) after the COBERS placement. Before the COBERS placement, the factors that were associated with students considering to work in a rural area were: extra allowance (OR = 0.2; 95% CI 0.1–0.6), and availability of social amenities (OR = 0.2; 95% CI 0.1–0.7). After their COBERS placement, the factors were: access to long distance courses (OR = 2.0; 95% CI 1.0–3.7) and being posted to a facility in a rural area (OR = 15.0; 95% CI 6.5–35.5). Before the COBERS placement the factors that influenced how long students thought they would be willing to work in a rural environment were: reliable electricity (IRR = 0.6; 95% CI 0.3–1.0) and Internet (IRR = 1.5; 95% CI 1.0–2.3), high salary (IRR = 0.4; 95% CI 0.3–0.7), and having skills to practice in rural settings (IRR = 2.0; 95% CI 1.3–3.1). Reliable electricity (IRR = 0.5; 95% CI 0.3–0.8) and long distance courses (IRR = 2.1; 95% CI 1.4–3.1) were significant motivators after having undergone the COBERS placement.

**Conclusions:**

The majority of health professions students do not intend to work in rural areas after they graduate. Improving the welfare of health professionals working in rural areas could attract more health professionals to rural areas thus addressing the maldistribution of health workers in Uganda.

## Background

In addition to a high disease burden, Uganda is faced with a severe shortage of health workers [1, 2]. Of the already insufficient doctors, a significant proportion emigrates [2]. Close to three-quarters of those that remain in Uganda mainly practice within urban areas [1]. The failure to attract and retain health workers leaves the hard-to-reach areas underserved yet the majority of the population lives in rural areas [3]. This mal-distribution of health workers constrains the overall health care system [4–6].

Several interventions to increase the number of doctors that graduate from the medical schools, to ensure their retention, as well as their equitable distribution to cater for the hard-to-reach areas have been proposed and implemented in sub-Saharan Africa including in Uganda. Among these are: salary supplements, recruiting medical students from rural areas and improving the infrastructure and social amenities in rural areas [1, 7–9]. However, these have not yet succeeded in combatting health workforce mal-distribution [5, 8, 10]. Community-Based Education (CBE) is one of the approaches being used to address the challenge of recruiting and retaining health workers in sub-Saharan Africa [11]. CBE has the potential to enhance the willingness of trainees to remain in their home countries after qualification and to practice in rural areas [11, 12].

The medical training institutions in Uganda under the Medical Education for Equitable Services for All Ugandans Consortium (MESAU) [13] implement Community-Based Education, Research and Services (COBERS) as a compulsory curriculum component [14]. The major goals of COBERS are to sensitize and acclimatize students to working in underserved communities and to enable them acquire the appropriate attitudes towards working in these areas [14]. However it has not been documented whether COBERS influences students’ attitudes towards working in Uganda and especially in underserved areas. Therefore the aim of this study was to explore the influence of COBERS on: (1) students’ attitudes towards working in rural areas, [15] factors associated with students’ intention to work in the rural areas, and [15] factors that influence the duration of time that the students intend to practice in rural areas after graduating.

### Medical education for equitable services to all Ugandans

Five medical universities in Uganda formed a consortium, Medical Education for Equitable Services to All Ugandans (MESAU), in 2010 in order to address challenges in health professions education [16]. These universities are: Makerere University College of Health Sciences (MakCHS), Mbarara University of Science and Technology (MUST), Gulu University (GU), Kampala International University (KIU) and Busitema University (BU) [17]. MESAU was established with funding from the US Government through the Medical Education Partnership Initiative (MEPI) and technical support from Johns Hopkins University [18]. MESAU aims to standardize medical education and develop the partner institutions into centers of excellence in medical education, research and service that address local and national needs to improve health in Uganda [16]. MESAU intends to produce health workers that are competent and well motivated to offer services that are locally relevant. The MESAU institutions have included Community-Based Education, Research and Service (COBERS) in their health professions curricula as one of the strategies to realize its overall aims [17].

### Community-Based Education Research and Services

Mbarara University of Science and Technology (MUST) at its inception in 1989 embraced CBE as the philosophy for health professions’ education within the Faculty of Medicine. In 2003, MakCHS introduced Community-Based Education and Services (COBES) into its curriculum [19] after a feasibility study carried out in 2000. The other medical universities in Uganda then followed suit. A component of research was added to the program, whereby the students identify a community problem for which they develop interventions. Having implemented the interventions, students also evaluate their impact [19]. COBERS is compulsory for medical, nursing, dentistry, pharmacy, biomedical engineering, cytotechnology, radiography and medical laboratory sciences students.

The program has different timing across the universities with MakCHS and KIU offering it once annually throughout the 5-year medical program, with each placement lasting 2–5 weeks [20]. Other universities offer the program once but for longer durations. The main components of COBERS are community health, community diagnosis, demography, communication skills, epidemiology, primary health care, biostatistics, health education and promotion, immunization, nutrition assessment and community engagement [21]. The services that students offer include: immunization, health education, patient clerkship, and community health promotion, and other community as well as health facility services as appropriate.

Each of the sites where students are placed has a site tutor who is a medically trained health worker at the hosting health facility and is responsible for facilitating and coordinating students’ learning activities and their assessment. Sites are assigned additional supervisors from the university who makes physical visits to the COBERS sites to monitor students’ progress. Throughout the COBERS placements, students are evaluated [22]. This evaluation comprises weekly assessment of activities entered into already structured logbooks. Further assessment is through evaluating performance in tutorial, which is held twice a week during the placement. At the end of the COBERS, there is a written examination paper that additionally contributes to the final mark. Other areas of assessment include reports submitted by students and oral presentations done by the students summarizing their activities [22].

## Methods

### Study design and study setting

This was a before-and-after study among undergraduate health professions students at four universities of the MESAU Consortium in Uganda namely: MUST, MakCHS, Gulu University and KIU. At the time of conducting the study, the Faculty of Health Sciences at Busitema University was not yet fully established. Faculty from the MESAU institutions developed a questionnaire with technical support from John-Hopkins University (JHU). The questionnaire was pre-tested at MUST with students who were not going for COBERS that academic year. We administered the questionnaire to 525 students in the 2011/2012 academic year in the month before they went for their first ever COBERS placement. The total number of students we had estimated from the university registers was 550. For Gulu in northern Uganda these were 4th year students, for KIU in South-western Uganda and MakCHS in central Uganda they were first year students and for MUST in South Western they were assorted. We administered the same questionnaire to 516 students after their first ever COBERS placement, which lasted 5–8 weeks. Students were invited to participate in the study through their respective student leaders and gathered at one location at each institution to complete the questionnaire before and after their COBERS placement. The questionnaire was distributed to the students who accepted to participate in the study and provided written informed consent.

### Study variables

We collected data on background characteristics including sex, institutional affiliation, year and program of study, area of residence during childhood and type of secondary school attended. For intended career choices after graduating and the factors that could influence these career choices, the tool included 28 positively worded items each with a 4-point Likert response scale (1 = strongly disagree to 4 = strongly agree). For intended career choices after graduating, the items included: practicing in the health sector, working in a rural area, working in Uganda, working in another African country, and working outside Africa. For factors influencing their intended career choice the items included: high salary, availability of reliable transport, electricity, security, infrastructure, medical supplies and equipment, Internet coverage, housing, good local schools for children, clean water and social amenities. Other factors included closeness to home area, ability to speak the local language, ability to interact with the university and other health professionals, being posted by the Ministry, being made in-charge of the health unit, access to long distance education courses, access to online medical literature, extra allowance, and having the necessary skills to practice in the rural areas. Information about the duration of time in completed years the students intended to practice in the rural areas was also collected.

### Data management and analysis

Data from all institutions was entered centrally into one Excel spreadsheet database at MakCHS, was checked for errors, and a copy was frozen. The data was exported to STATA version 12 for analysis. Data was adjusted for clustering at institution level. Analysis was stratified to before and after COBERS placement. Descriptive data were summarized and presented as means for continuous data and percentages for the categorical data.

Likert-scale values for each item in the tool were categorized into a binary variable. ‘Strongly disagree’ and ‘Disagree’ were grouped together as ‘Disagree’ while ‘Agree’ and ‘Strongly agree’ were grouped into a single variable ‘Agree’. Comparisons were made between the two generated groups before and after the COBERS placement using the McNemar’s Chi square test. Significance was set at a p value of 0.05 or less.

For all the mathematical models, factors from the Likert scale were dichotomized as described above. For each of the factors, a model was run for the students before the COBERS placement and another model for after the placement. To assess the factors that influence the students’ intention to work in rural areas, the outcome was dichotomized as ‘Disagree’ or ‘Agree’ to work in rural areas. A logistic regression model was then run. Odds ratios (OR) were the measure of association used. For the factors influencing the number of years of practicing in rural areas, the outcome was a count in completed years. A Poisson regression model was employed after assessing and ruling out suitability for negative binomial model. Incidence rate ratio [15] was used as measure of association. For all the models only factors with p values of 0.1 or less at bivariate analysis were entered in a multivariate model. Confounding factors were assessed at a 10% difference between the measure of association for the unadjusted and adjusted models.

## Results

A total of 525 students participated in the survey before COBERS while 516 students participated in the survey following the community placement. A few questionnaires had some missing information such as gender and year of study.

### Socio-demographic characteristics

Of the 525 students who participated in the survey before their first COBERS placement, the majority were males, 355/510 (69.6%). MakCHS had the most students, 194/525 (37.0%). Most students were doing medicine, 283/514 (55.1%) and were in their first year of study, 301/515 (58.5%). Details are shown in Table 1. Comparable proportions were seen among the students that participated in the survey following their COBERS placement. The mean duration of time the students intended to practice in rural areas was 2.6 ± 3.3 years with median 2 (1, 3) years.

**Table 1:** Background characteristics of undergraduate health professions students in Uganda’s medical schools, 2012

Variable	Before COBERS (n = 525)	After COBERS (n = 516)
	N (%)	N (%)
University		
Gulu university	32 (6.1)	34 (6.6)
Kampala Int. University	169 (32.2)	165 (32.0)
Makerere University	194 (37.0)	140 (27.1)
Mbarara University	130 (24.8)	177 (34.3)
Degree program^a^		
Dentistry	11 (2.1)	6 (1.2)
Laboratory technology	70 (13.6)	46 (9.2)
Medicine	283 (55.1)	316 (62.8)
Nursing	35 (6.8)	35 (7.0)
Pharmacy	88 (17.1)	59 (11.7)
Others	27 (5.3)	41 (8.2)
Year of study^a^		
Year one	301 (58.5)	341 (66.9)
Year two	40 (7.8)	33 (6.5)
Year three	82 (15.9)	46 (9.0)
Year four	83 (16.1)	76 (14.9)
Year five	9 (1.8)	14 (2.8)
Sex^a^		
Male	355 (69.6)	339 (69.3)
Female	155 (30.4)	150 (30.7)
Residence in childhood^a^		
Kampala	154 (29.8)	183 (35.9)
Municipalities out of Kampala	187 (36.2)	157 (30.8)
Rural Uganda	163 (31.6)	159 (31.2)
Other country	12 (2.4)	11 (2.2)
Type of secondary school^a^		
Boarding school	382 (73.6)	389 (75.7)
Urban day school	94 (18.1)	85 (16.5)
Rural day school	43 (8.3)	40 (7.8)
Choice of destination^a^		
Urban	290 (56.0)	261 (51.6)
Rural	228 (44.0)	245 (48.4)

*COBERS* Community-Based Education, Research and Services

^a^Some of the participants had missing information

### Effect of COBERS on attitudes towards work in rural areas

Before COBERS, 228/518 (44.0%; 95% CI 39.7–48.3%) indicated that they intended to work in rural areas as compared to 245/506 (48.4%; CI 44.1–52.8%) after the COBERS placement (p = 0.03). The proportion of students intending to work in the health profession after graduation reduced from 493 (95.5%) before COBERS to 462 (91.1%) after COBERS (p = 0.005). The proportion of students that reported the following social and professional factors as influencing them to work in rural areas reduced following COBERS placement: reliable transport from 472 (90.6%) to 441 (86.6%), p value 0.046; reliable electricity from 464 (88.9%) to 415 (82.7%), p value 0.04; good security from 498 (95.2%) to 459 (90%), p value 0.001; good housing from 451 (87.1%) to 409 (82.6%), p value 0.049; high salary from 430 (82.9%) to 361 (71.5%), p value 0.01; access to medical literature from 372 (71.5%) to 332 (65.4%), p value 0.033 and having the necessary skills in community health from 447 (85.8%) to 400 (80.2%), p value 0.016. Details are highlighted in Table 2.

**Table 2:** Influence of COBERS on the attitudes of undergraduate health professions students in Uganda towards working in rural areas, 2012

	Before COBERS		After COBERS		p value
	N	Score	N	Score	
Choice of career destination					
I intend to work in a rural area	518	2.24	506	2.34	*0.082*
I intend to work in a health profession after I graduate	516	3.52	507	3.36	*<* *0.001*
I intend to work in a Uganda after I graduate	358	3.04	336	2.88	*0.006*
I intend to work in another African country after I graduate	518	2.58	497	2.49	0.110
I intend to work outside Africa after I graduate	509	2.61	499	2.48	0.053
Factors influencing intentions to work in the rural areas					
Social attributes					
Reliable transport	521	3.43	509	3.22	*<* *0.001*
Reliable electricity	522	2.24	502	3.17	*0.001*
Availability of clean water	231	3.48	341	3.27	*<* *0.001*
Good security	523	3.57	510	3.38	*<* *0.001*
Good infrastructure	518	3.26	510	3.13	*0.003*
Reliable internet	522	2.97	506	2.85	*0.029*
Good housing	518	3.22	495	3.11	*0.044*
Availability of good local schools for my children	521	3.05	507	2.93	*0.029*
Working close to where I grew up	524	2.06	508	2.09	0.430
Being able to speak the local language	521	2.78	505	2.74	0.560
High salary	519	3.23	505	2.93	*<* *0.001*
Professional attributes					
Being able to interact with a university	518	2.60	506	2.55	0.365
Being able to interact with other health professionals	521	3.50	507	3.28	*<* *0.001*
Being posted in the rural area	516	2.33	500	2.46	*0.016*
Being made the in-charge of the hospital	517	2.36	499	2.37	0.769
Having access to long distance education courses	507	2.65	501	2.53	*0.035*
Having access to electronic medical literature	520	2.86	508	2.69	*0.002*
Being given extra allowance to work in rural areas	517	2.95	508	2.80	*0.010*
Having specific skills in community health	521	3.27	499	3.06	*<* *0.001*
Having adequate medical supplies and equipment	234	3.39	339	3.21	*0.033*

*COBERS* Community-Based Education, Research and Services

### Factors influencing the decision to work in the rural areas

As shown in Table 3, before undertaking the COBERS placement, the factors that were associated with intention to work in a rural area at multivariate analysis were: geographic area (urban or rural) where student spent his or her childhood, access to long distance medical courses (OR = 2.3; 95% CI 1.0–5.8), receiving an extra allowance to work in the rural areas (OR = 0.2; 95% CI 0.1–0.6), being posted by the Ministry (OR = 4.8; 95% CI 2.3–9.7) and availability of social amenities (OR = 0.2; 95% CI 0.1–0.7).

**Table 3:** Factors associated with intention to work in the rural areas as expressed by undergraduate health professions students prior to having a COBERS placement in 2012

Variables	Bivariate analysis			Multivariate analysis		
	cOR	95% CI	p value	aOR	95% CI	p value
Sex						
Male	1			1		
Female	0.6	0.4–0.9	0.008	1.3	0.5–3.3	0.587
Childhood home						
Kampala	1			1		
Urban out of Kampala	3.6	2.2–5.8	0.001	3.5	1.1–11.3	*0.034*
Rural Uganda	6.6	4.0–10.9	0.001	4.7	1.5–15.0	*0.010*
Other country	3.8	0.5–27.6	0.195	3.1	0.1–4.5	0.328
Response is disagree	1			1		
High salary	0.5	0.3–0.8	0.005	0.5	0.1–2.2	0.373
Being posted in rural area	7.4	4.5–12.3	0.001	4.8	2.3–9.7	*0.001*
Access to long distance courses	1.7	1.2–2.4	0.005	2.3	1.0–5.8	*0.050*
Extra allowance	0.4	0.3–0.7	0.001	0.2	0.1–0.6	*0.004*
Availability of social amenities	0.3	0.1–0.6	0.001	0.2	0.1–0.7	*0.012*

After the COBERS placement at multivariate analysis the factors that were significantly associated with choice to work in the rural areas included: access to long distance courses (OR = 2.0; 95% CI 1.0–3.7), and being posted in the rural areas by the ministry (OR = 15.0; 95% CI 6.5–35.5). Details are shown in Table 4.

**Table 4:** Factors associated with intention to work in rural areas as expressed by undergraduate health professions students after a 5–8 weeks COBERS placement in 2012

Variables	Bivariate analysis			Multivariate analysis		
	cOR	95% CI	p value	aOR	95% CI	p value
University						
Gulu	1			1		
MakCHS	0.9	0.4–1.9	0.708	0.3	0.1–1.1	0.062
MUST	1.7	0.8–3.8	0.191	0.6	0.2–1.9	0.387
Sex						
Male	1			1		
Female	0.8	0.6–1.2	0.299	1.0	0.5–1.9	0.988
Response is disagree	1			1		
High salary	0.5	0.3–0.8	0.001	0.6	0.2–1.4	0.216
Good housing	0.6	0.4–1.0	0.049	0.5	0.2–1.2	0.126
Being posted in rural area	11.4	7.0–18.5	0.001	15.1	6.5–35.5	*0.001*
Access to long distance courses	2.2	1.6–3.2	0.001	2.0	1.0–3.7	*0.039*
Having the skills needed	2.7	1.7–4.2	0.001	1.3	0.6–2.8	0.506

### Factors influencing intended duration of work in rural areas

Before the COBERS placement, at multivariate analysis the factors that influenced the intended duration of work in the rural areas included: being female (IRR = 0.6; 95% CI 0.4–0.9), attending urban secondary school (IRR = 0.5; 95% CI 0.3–0.8), availability of reliable electricity (IRR = 0.6; 95% CI 0.3–1.0) and Internet (IRR = 1.5; 95% CI 1.0–2.3), being paid high salary (IRR = 0.4; 95% CI 0.3–0.7), and having the necessary skills to practice in a rural setting (IRR = 2.0; 95% CI 1.3–3.1) as shown in Table 5.

**Table 5:** Factors influencing intended duration of working in rural areas (after graduation) by undergraduate health professions students that have not had a COBERS placement, 2012

Variable	Bivariate analysis			Multivariate analysis		
	cIRR	95% CI	p value	aIRR	95% CI	p value
Sex						
Male	1			1		
Female	0.6	0.5–0.8	0.001	0.6	0.4–0.9	*0.023*
School attended						
Boarding school	1			1		
Urban day school	0.5	0.3–0.8	0.008	0.5	0.3–0.8	*0.008*
Rural day school	1.0	0.6–1.7	0.998	1.0	0.6–1.7	0.998
Response is disagree	1			1		
High salary	0.5	0.4–0.6	0.001	0.4	0.3–0.7	*0.001*
Reliable electricity	0.8	0.7–1.0	0.012	0.6	0.3–1.0	*0.040*
Reliable internet	1.2	1.1–1.4	0.003	1.5	1.0–2.33	*0.050*
Having the skills needed	1.4	1.1–1.8	0.003	2.0	1.3–3.1	*0.002*

Table 6 shows the factors associated with intended duration of work in the rural areas after students had undergone a COBERS placement. These included university attended, availability of reliable electricity (IRR = 0.5; 95% CI 0.3–0.8), access to long distance medical courses (IRR = 2.1; 95% CI 1.4–3.1), and having the intention to work in another African country (IRR = 0.5; 95% CI 0.4–0.8).

**Table 6:** Factors influencing intended duration of working in rural areas after graduating by undergraduate health professions students that have undergone a 5–8 weeks COBERS placement in 2012

Variable	Bivariate analysis			Multivariate analysis		
	cIRR	95% CI	p value	aIRR	95% CI	p value
University						
Gulu	1			1		
KIU	0.8	0.6–1.0	0.063	–		
MakCHS	0.4	0.3–0.6	0.001	0.5	0.3–0.7	*0.001*
MUST	0.4	0.3–0.6	0.001	0.3	0.1–0.4	*0.001*
Response is disagree	1			1		
Reliable electricity	0.6	0.5–0.7	0.000	0.5	0.3–0.8	*0.005*
Access to long distance courses	1.1	1.0–1.3	0.071	2.1	1.4–3.1	*0.001*
I intend to work in another African country	0.7	0.6–0.8	0.001	0.5	0.4–0.8	*0.001*

## Discussion

This study assessed health professions students’ intentions to practice in a rural environment, their intended duration of practice in this setting, the factors influencing their intentions and the effect of a COBERS placement during their training on their intentions. Our study is unique in that it included all established medical training institutions in Uganda at the time. We found that both before and after a COBERS placement lasting 5–8 weeks, less than half of the students expressed intentions to practice in rural areas. However, the proportion of students who expressed intentions to practice in rural areas increased following the COBERS placement. Even though we only assessed intentions, the findings from this study are in parallel with findings about the actual maldistribution of health professionals in low-income countries [23].

### Factors influencing choice to practice in the rural areas

Before COBERS, the students that grew up in Kampala, the capital city of Uganda, were more than three times willing to work in the rural areas compared to students who grew up in municipalities beyond Kampala and in the rural areas. A similar finding was reported in Ghana where an overwhelming majority of graduates from the towns and cities were willing to work in the rural areas [24]. This could be due to a desire to serve as a gesture of compassion [6]. In contrast, a study in eight low-to-middle-income countries found that students who had prolonged exposures to rural settings were the ones more likely to express intentions towards a rural career [23].

The findings demonstrate a change in students’ attitudes towards work in rural areas after the COBERS placement in that they are less influenced by the social amenities than they were before COBERS. This implies that COBERS has the potential to increase on the number of students likely to work in rural areas by changing their attitudes regarding the relative importance of social amenities as an influencing factor for their decisions. Having opportunities for long distance courses positively influenced students’ intention to work in rural areas. Additionally, students expressed more willingness to practice in rural areas if the Ministry posted them there. These findings are in parallel with previous studies [25, 26]. In contrast, receiving extra allowances and availability of social amenities in the rural areas negatively impacted the students’ intention to practice in the rural areas. This demonstrates that some students career preferences are not centered on remuneration but on their passion and the need to pay back to society [26].

### Factors influencing the duration students are willing to practice in rural areas following graduation

Before the COBERS rotation, being female, attending an urban day school as opposed to boarding school, and being offered a high salary were associated with a reduction in the intended duration of working in the rural setting. Students that have not had rural exposure have negatively biased views regarding practicing in remote settings [6]. Just like findings from other studies, females are less likely to stay and practice in rural areas for long. This could be explained by prevailing cultural values where by women follow their spouses on their job placements [6].

Availability of reliable Internet and having the necessary skills were associated with a higher intended duration of working in rural areas. After the COBERS placement, unreliable electricity was associated with a reduction in the intended duration of working in the rural areas. The majority of students are youths who use mobile technology and computers for academics as well as recreation. This accounts for reliable electricity and reliable Internet being a major influence on intended career location [26]. Having access to long distance courses was associated with significantly higher intended duration of practicing in the rural areas. Students who had the intention of working in another African country were more likely to spend 50% less time working in the rural areas as compared to their counterparts who did not express the intention to work in another African country [25].

### Limitations

The majority of students took the survey both before and after COBERS, which could have created dependence. This was however accounted for by applying methods for dependence during data analysis and performing stratified analysis for the study population before and after COBERS placement. We did not link individual students’ before and after interviews but rather used aggregated responses. Since we did not achieve maximum response rate, we cannot ascertain overlap in the before and after responses per individual. However, the non-response rates were considerably low for both interviews which meant that the overlap was high as would be desired. The study assessed future intentions, which may not necessarily result into the same actions after graduation. The researchers relied on self-report and this is prone to social desirability biases. Our study assessed student’s intentions after a short period, 5–8 weeks of COBERS exposure. It is not known whether a longer single exposure or multiple exposures during the course of their training will lead to different results. This could be ascertained when follow-up studies are conducted. The data was aggregated across all years of study among the different universities. First year students are likely to have different knowledge and experience, which could confound the findings. In our study, we did not employ qualitative research methods. These would add more valuable information in understanding students’ decisions to work in rural areas. Given that all health professions students at the MESAU institutions all have to undergo COBERS placements as part of the approved curricula, we did not have a control group. This would have added more information regarding the influence of COBERS.

## Conclusions

The majority of health professions students do not intend to work in rural areas after they graduate; however, there was a slight increase in the proportion of students expressing intentions to work in rural areas after they graduate. Going by the factors that influenced the students’ intentions, improving the welfare of health professionals working in rural areas could attract more health professionals to rural areas thus addressing the mal-distribution of health workers in Uganda.

## Abbreviations

COBERS: Community-Based Education, Research and Services
HIV: Human Immunodeficiency Virus
IRR: incident rate ratios
JHU: Johns-Hopkins University
KIU: Kampala International University
MakCHS: Makerere University College of Health Sciences
MUST: Mbarara University of Science and Technology
OR: odds ratio
UNCST: Uganda National Council for Science and Technology
